# Case of Poisoning by Strychnia—Case of Extra-Uterine Pregnancy

**Published:** 1866-11

**Authors:** S. T. Clark


ART. IV—*Case of Poisoning by Strychnia—Case of Extra-Uterine Pregnancy.* BY S. T. CLARK, A. M., M. D.

R. H., aged 30, married, having stimulated to excess for five days, was seized with symptoms of *delirium tremens.* The memory of sufferings he once witnessed in a victim to this malady, together with the maddening effects of the disease, prompted him, at 6½ P. M., May 16th, to go to a neighboring druggist and procure a drachm of strychnia, which being done at 7½ o'clock he entered his own cellar and drawing a glass of ale, added half the strychnia and swallowed the draught.

The very tonic effect of the dose seems to have changed his mind on the subject of suicide, for he immediately sought his wife and reported his condition; ordered mustard and water at once, and desired that I might be sent for. Mrs. H. knowing what had been the history of the case for the past week, discredited him and remonstrated with him for trifling with her fears, and refused to administer the emetic or send for a physician; in the midst of the dispute he fell to the floor in a spasm. A messenger was despatched for me; the mustard and water prepared, which H. swallowed as soon as the spasm passed.

On my arrival, I found he had vomited after taking the mustard and had been convulsed almost constantly since the emesis. He was lying quietly when I entered the room, but the pressure of my fingers on his wrist, provoked the most frightful spasm I ever witnessed. The entire muscular system was thrown into such rapid and violent motion, that the bed on which he lay trembled, until every article of furniture in the room, and even the floor under us, was shaken. The face was black and swollen, the eyes started from their sockets, the tongue protruding and blackened. In fact, I did not believe he would survive this spasm, which must have continued nearly five minutes, and ended in asphyxia, from which the patient gradually passed into nearly the same condition as that in which I first found him.

On learning the facts in the case, I had sent for chloroform and sulphate of zinc; both articles were now delivered, and without delay I administered zinci sulph. grs. xl, in solution. The act of swallowing was followed by spasm, but its severity was greatly moderated by the inhalation of the chloroform. Having decided to try the full effects of anæsthesia, the use of the chloroform was continued from this time (8 P. M.) until 4 o'clock next morning, with frequent attempts to suspend its use, but as soon as the napkin was removed the spasm re-appeared.

At half past eight the zinc was ejected with some very dark fluid and a small quantity of fresh blood.

At 4 A. M. the patient manifested great uneasiness and moaned occasionally. I withheld the chloroform and found his suffering was from distention of the bladder; he voided a very large quantity of almost colorless urine. The withdrawal of the bed-pan provoked a slight spasm. I now administered a half bottle of salad oil, and as soon as he had taken it he was again convulsed. From this time until 12 M. he was held in a complete state of anæsthesia.

From 11 o'clock until 12 he sweat most profusely, and manifested marked signs of prostration and the chloroform was withheld, no spasms recurred, and with the exception of soreness and weakness he was comfortable. The muscles of the throat and back were partially paralyzed, and the surface of the body had the appearance of having been beaten with rods; as soon as this discoloration passed away the function of the muscles was resumed. His recovery was constant and rapid. The quantity of strychnia swallowed was more than 20 grains.* The amount of chloroform used was 1½ lbs.

* Recovery after taking three or four grains of strychnia is mentioned by Stille. Dr. H. G. Thomas reports recovery after taking five grains, and Dr. S. S. Harris reports two cases after a dose of from six to eight grains. Lemery, while the real

*Case of Extra-Uterine Pregnancy.*—June 26th, I was called to see Mrs. Dr. H., aged 33, mother of four children; found her pale and cold, pulse almost wanting, and suffering intense spasmodic pain in the region of the uterus, with occasionally a severe pain in the arms and right leg. She was inhaling sulphuric ether with good effect. I ordered morphia and brandy to be taken and mustard applied to the abdomen. In less than an hour perfect reaction was established and I left my patient sleeping.

Saw her next morning, and learned she had been flowing moderately for a week, and prior to that time she had not menstruated for seven weeks. She appeared very comfortable, only complaining of occasional pain in the limbs.

On the 28th saw Mrs. H., early in the morning, was feeling well, save nausea, which she had suffered every morning for the past five days. Ordered her to take five grains of sub nit. bismuth with one of oxalate of cerium, every morning. The flow still continuing, ordered

℞ Acidi gallici, - - - - ʒiv.

Glycerine, - - - - - ℥i.

Aqua rosæ, - - - - ℥ii. M.

A teaspoonful to be taken every four hours. A decided periodicity having been noticed in the pain of the limbs and abdomen, gave ℞ Hyoscyami ext. - - - - ʒi.
Quin. valerianatis, - - grs. xxx. M.
Fiat pilulas, No. xxx.

One pill to be taken morning, noon and night.

July 1st. Had been free from pain since taking the pills; nausea continued; gave creasote one drop to a fluid ʒ of water—*pro re nata.* Patient continued to improve, nausea relieved by the creasote, appetite good, rode out on the 7th and 8th.

Monday, 9th, at 6 P. M., was hastily summoned to her bed-side to find her in much the same condition as on the 26th of last

---

properties of this drug were unknown, asserted that it was not poisonous to man. Recently the general use of nux vomica, especially the active principles of it. establish its poisonous properties, and when recovery takes place after a dose of twenty or thirty grains, the purity of the specimen demands attention. It is greatly liable to adulteration. Chloroform is said to be antidotal administered internally, and many other substances are said to counteract its effects; it is, however, probable that nothing but death would arrest the symptoms after the administration of twenty or thirty grains of the pure alkoloid.—Ed.

month, but paler, colder and more nearly pulseless. Had a spasm of most excruciating pain at 3 P. M. while in her chair, followed by the palor, etc., etc.

At 7 o'clock ether and morphia failing to alleviate her suffering, and the prostration increasing, I called Dr. McCollum, who kindly remained with me until two o'clock the next morning, when the patient died.

The afternoon of the 10th, at my request, Drs. Gould and Babbitt, in the presence of several other medical gentlemen, made a *post mortem* examination. Rigor mortis, well marked; unusual bloodless appearance of fingers and lips, unnatural fullness of abdomen. *Sectio cadaver* revealed plentiful adipose deposit; and the peritoneal cavity being opened a large quantity of fluid blood escaped, after which two or more quarts of clotted blood were removed, and under these clots, lying upon the small intestines, was a fœtus of about ten weeks' growth, membranes unruptured.

The pregnancy was *ovario-tubal;* the right ovary was enlarged to twice its normal size, and the fimbriated extremity of the fallopian tube converted into a sac, helped to form the receptacle for the fœtus. The ovary was changed in its structure, having become like a bundle of blood-vessels, and the tube from the sac was imperforate. There appeared to have been a bursting of the ovary, as well as a separation of the tubal sac *from* the ovary, from which the hemorrhage took place. The placenta was external to the ovary, attached to it and the surrounding viscera. The uterus was somewhat enlarged and contained the *decidua.*

---

A New Instrument for Subcutaneous Injections.—M. Bouilland lately presented to the Academy of Medicine of Paris an invention of M. Dancet, consisting of a hollow needle adapted to a metallic tube, ending in a small cup covered with an india-rubber membrane. By slight pressure upon the latter the fluid is injected into the areolar tissue, and a simple mechanism within the cup allows of the counting of the drops injected. Another and simpler needle on the same principle may be used for vaccination.

